# Berberine Reduces Fibronectin Expression by Suppressing the S1P-S1P2 Receptor Pathway in Experimental Diabetic Nephropathy Models

**DOI:** 10.1371/journal.pone.0043874

**Published:** 2012-08-24

**Authors:** Kaipeng Huang, Weihua Liu, Tian Lan, Xi Xie, Jing Peng, Juan Huang, Shaogui Wang, Xiaoyan Shen, Peiqing Liu, Heqing Huang

**Affiliations:** 1 Laboratory of Pharmacology & Toxicology, School of Pharmaceutical Sciences, Sun Yat-sen University, Guangzhou, China; 2 Guangzhou Institute of Cardiovascular Disease, The Second Affiliated Hospital of GuangZhou Medical University, Guangzhou, China; 3 Vascular Biology Research Institute, Guangdong Pharmaceutical University, Guangzhou, China; Kyushu Institute of Technology, Japan

## Abstract

The accumulation of glomerular extracellular matrix (ECM) is one of the critical pathological characteristics of diabetic renal fibrosis. Fibronectin (FN) is an important constituent of ECM. Our previous studies indicate that the activation of the sphingosine kinase 1 (SphK1)-sphingosine 1- phosphate (S1P) signaling pathway plays a key regulatory role in FN production in glomerular mesangial cells (GMCs) under diabetic condition. Among the five S1P receptors, the activation of S1P2 receptor is the most abundant. Berberine (BBR) treatment also effectively inhibits SphK1 activity and S1P production in the kidneys of diabetic models, thus improving renal injury. Based on these data, we further explored whether BBR could prevent FN production in GMCs under diabetic condition via the S1P2 receptor. Here, we showed that BBR significantly down-regulated the expression of S1P2 receptor in diabetic rat kidneys and GMCs exposed to high glucose (HG) and simultaneously inhibited S1P2 receptor-mediated FN overproduction. Further, BBR also obviously suppressed the activation of NF-**κ**B induced by HG, which was accompanied by reduced S1P2 receptor and FN expression. Taken together, our findings suggest that BBR reduces FN expression by acting on the S1P2 receptor in the mesangium under diabetic condition. The role of BBR in S1P2 receptor expression regulation could closely associate with its inhibitory effect on NF-**κ**B activation.

## Introduction

Renal fibrosis, including glomerulosclerosis and tubulo-interstitial fibrosis, is one of the major pathological changes caused by progressive diabetic nephropathy (DN). It is characterized by glomerular mesangial cells (GMCs) proliferation, excessive accumulation of extracellular matrix (ECM) proteins, mesangial expansion and thickening of the glomerular basement membrane in the early stage, as well as glomerulosclerosis and tubulo-interstitial fibrosis in the late stage, which eventually results in the loss of renal function [1], [2]. GMCs are some of the inherent cells of glomeruli. Changes in the quantity and physiological function of GMCs, including phagocytosis, scavenge dysfunction, as well as excessive synthesis and secretion of ECM, play important roles in the pathological development of DN [3], [4]. We have previously reported that high glucose (HG) stimulated GMCs proliferation and ECM component-fibronectin (FN) expression leading to ECM deposition, which initiated and accelerated the pathological progression of DN [5]. Therefore, the intervention of ECM synthesis or promotion of its degradation in GMCs with the subsequent prevention and delay of renal fibrosis would be greatly significant in DN treatment [6].

Sphingosine 1-phosphate (S1P) is a bioactive sphingolipid metabolite synthesized exclusively from sphingosine kinases (SphKs) [7]. S1P can act both intracellularly as a second messenger and extracellularly as a ligand for its specific receptors-S1PRs, to activate or inhibit diverse signaling pathways. Consequently, S1P mediates important physiological processes such as cell migration and cytoskeleton rearrangements [8]–[10]. Numerous studies have showed that S1PRs are closely associated with the development of multiple human diseases, such as cancer, atherosclerosis, and inflammation [11]–[13]. However, the roles of S1PRs in diabetes and diabetic complications are still not fully understood. Researchers exploring the mechanisms of S1PRs in diabetes mainly focus on immune regulation, inflammation, and angiogenesis [14], [15]. Both our previous *in vivo* and *in vitro* experiments have revealed that the SphK1-S1P signaling pathway is activated in the kidneys and GMCs under diabetic condition, concomitant with elevated FN production. The intervention of SphK1 and reduction of S1P level reverse the increase in FN expression in GMCs. We have also found that the sphingosine 1-phosphate receptor 2 (S1P2 receptor) is expressed dominantly among the five S1PRs in the above-mentioned animal and cell models, suggesting that the SphK1/S1P/S1P2 receptor pathway plays a key regulatory role in the pathological progression of DN [16]–[18].

Berberine (BBR; [C_20_H_18_NO_4_]^+^) is an isoquinoline alkaloid isolated from *Coptidis rhizome* and *Cortex phellodendri*. Recent studies have shown that BBR has potential clinical application as a therapeutic drug for diabetes and diabetic complications. BBR also has pharmacological characteristics, such as multi-targets, extensive effects, and complicated mechanisms [19]–[24]. However, the underlying mechanisms responsible for the renal protective role of BBR in DN have not yet been elucidated. Our recent studies have shown that BBR ameliorates renal function by reducing SphK1 expression and activity, as well as the S1P level in alloxan-induced diabetic mice kidneys [16]. Based on these data, further investigations are needed to verify whether BBR prevents FN production under diabetic condition via the S1P2 receptor.

## Results

### Effects of BBR on Metabolic and Biochemical Parameters in STZ-induced Diabetic Rats

Compared with normal rats, streptozotozin (STZ)-induced diabetic rats exhibited the following typical symptoms of diabetes: “three more and one less” (more eating, drinking, and urine, but less body weight), obviously increased fasting blood glucose (FBG) levels, significantly diminished body weight, and augmented kidney weight/body weight ratio (KW/BW). When the experiment was terminated on the 12th week, all blood urea nitrogen (BUN), serum creatinine (Cr), and 24 h albuminuria (UP 24 h) increased (*P*<0.05, Table. 1) in diabetic rats. This result suggested the emergence of diabetic renal dysfunction. In contrast, the rats in the BBR treatment group showed improved typical symptoms of diabetes as well as obviously reduced FBG, KW/BW, BUN, Cr, and UP 24 h levels (*P*<0.05, Table. 1). This finding indicated that BBR effectively ameliorated the renal function of diabetic rats.

**Table 1:** Effects of BBR on metabolic and biochemical parameters in STZ-induced diabetic rats.

Items	Normal	Diabetes	Diabetes+Berberine
Blood glucose (mM)	5.14±0.18	27.78±5.55*	16.70±3.25* #
Body weight (g)	426.29±13.30	165.00±6.00*	203.50±15.03* #
Kidney weight (g)	2.57±0.09	2.58±0.19	2.22±0.11
KW/BW (%)	0.60±0.02	1.56±0.09*	1.10±0.04* #
BUN (mM)	5.37±0.44	34.58±3.60*	12.35±2.98#
Cr (µM)	30.00±2.18	92.13±16.64*	42.25±5.45#
UP 24h (g/L)	0.011±0.002	0.112±0.026*	0.079±0.014*

Data are represented as Means ± SDs, BUN: blood urea nitrogen, Cr: serum creatinine, UP 24 h: urine protein for 24 hours,

* *P*<0.05 vs. normal,

# *P*<0.05 vs. diabetes.

### BBR Reduced the mRNA and Protein Expression of S1P2 Receptor in Diabetic Rat Kidneys

**Figure 1:**
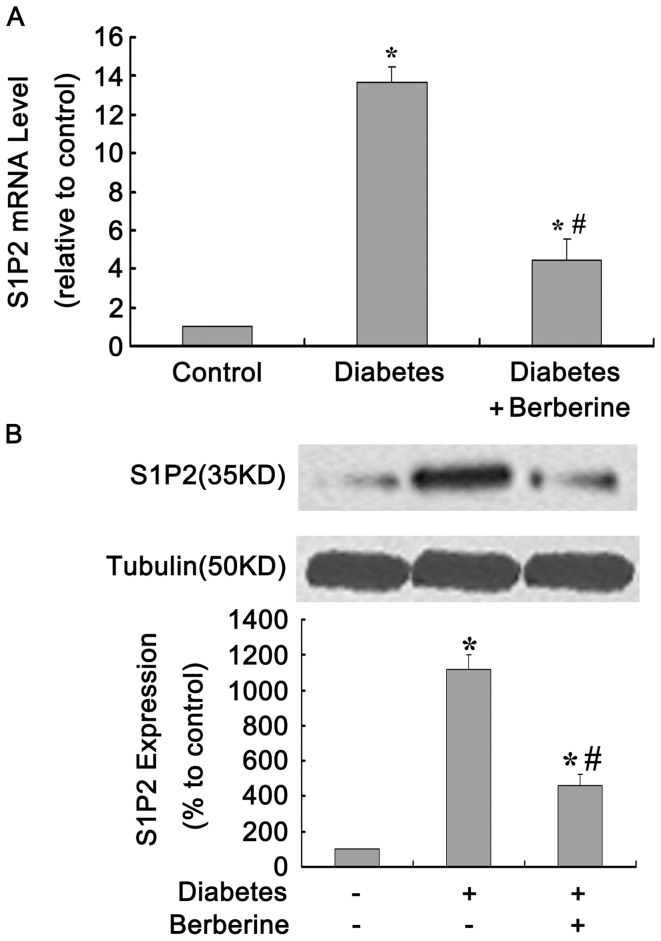
BBR reduced mRNA and protein expression of S1P2 receptor in diabetic rat kidneys. STZ-induced diabetic rats were treated for 12 weeks with BBR, and the levels of mRNA (A) and protein (B) of S1P2 receptor were evaluated by real-time PCR and Western blot, respectively. Data are represented as Means ± SDs, *P<0.05 vs. control, #*P*<0.05 vs. diabetes.

Compared with the control group, the mRNA level of S1P2 receptor in diabetic rat kidneys increased approximately 13-fold (*P*<0.05). In the BBR treatment group, this level was reduced by ∼65% compared with that in diabetic models (*P*<0.05, Fig. 1 A). The protein expression of S1P2 receptor were detected by Western blot assay and the data showed that the changes in S1P2 receptor protein levels in the diabetic and BBR treatment groups were similar to those of mRNA. In contrast to the control, S1P2 receptor protein levels in diabetic rat kidneys were enhanced 11-fold (*P*<0.05), whereas BBR treatment reduced the S1P2 receptor protein levels by ∼63% compared with those in diabetic models (*P*<0.05, Fig. 1 B).

### BBR Reduced FN Expression in Diabetic Rat Kidneys

**Figure 2:**
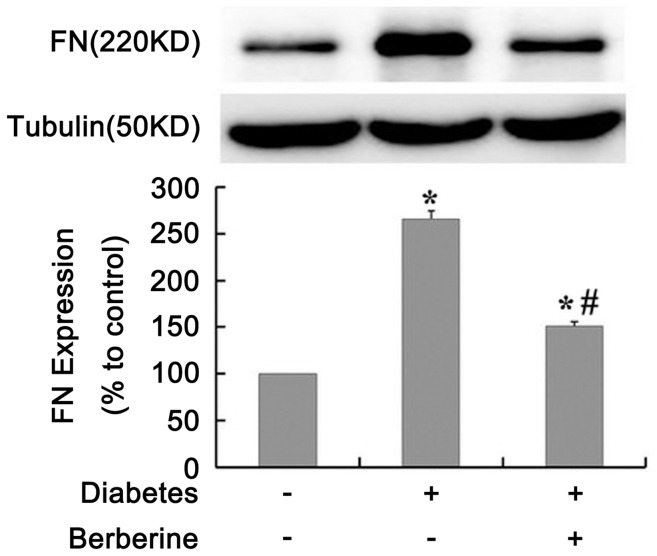
BBR reduced FN expression in diabetic rat kidneys. STZ-induced diabetic rats were treated for 12 weeks with BBR, and the levels of FN were detected by Western blot assay. Data are represented as Means ± SDs, *P<0.05 vs. control, #*P*<0.05 vs. diabetes.

FN is one of the major components of ECM in GMCs. FN overproduction reflects the serious extent of glomerularsclerosis to some degree. Therefore, we also examined the effects of BBR on FN protein expression in diabetic rat kidneys. The protein levels of FN in diabetic rat kidneys increased three-fold compared with those in the normal group, and were reduced to half by BBR treatment (*P*<0.05, Fig. 2).

### Effects of BBR on S1P2 Receptor Expression and Distribution in GMCs under HG Condition

**Figure 3:**
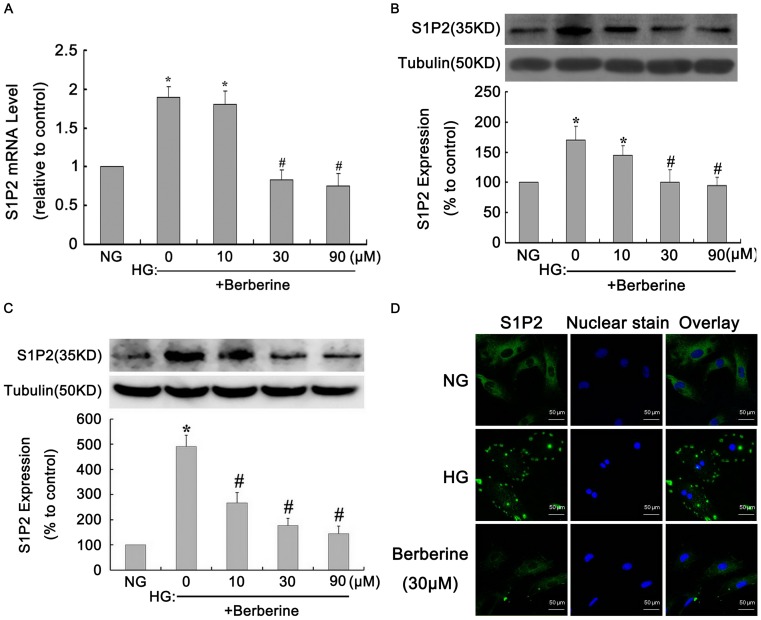
Effects of BBR on S1P2 receptor expression in GMCs under HG condition. GMCs were treated with different dose of BBR under HG condition. After 24 h of incubation, the levels of mRNA (A) and protein (B) of S1P2 receptor were evaluated using real-time PCR and Western blot, respectively. Expression (C) and distribution (D) of S1P2 receptor on the membrane of GMCs was detected by Western blot and LSCM. Data are represented as Means ± SDs, *P<0.05 vs. normal glucose (NG), #*P*<0.05 vs. HG.

Both mRNA and protein levels of S1P2 receptor were markedly elevated by 30 mM HG treatment for 24 h in GMCs (*P*<0.05), BBR treatment inhibited the increase in S1P2 receptor expression in a dose-dependent manner (*P*<0.05, Figs. 3 A and 3 B). Western blot assay and Laser scanning confocal microscopy (LSCM) data indicated that S1P2 receptor expression and distribution were obviously increased on the cell membrane of GMCs under hyperglycemic condition (*P*<0.05), and that BBR significantly reduced S1P2 receptor levels on the cell membrane (*P*<0.05, Figs. 3 C and 3 D).

### BBR Suppressed S1P2 Receptor Mediated FN Expression in GMCs under HG Condition

**Figure 4:**
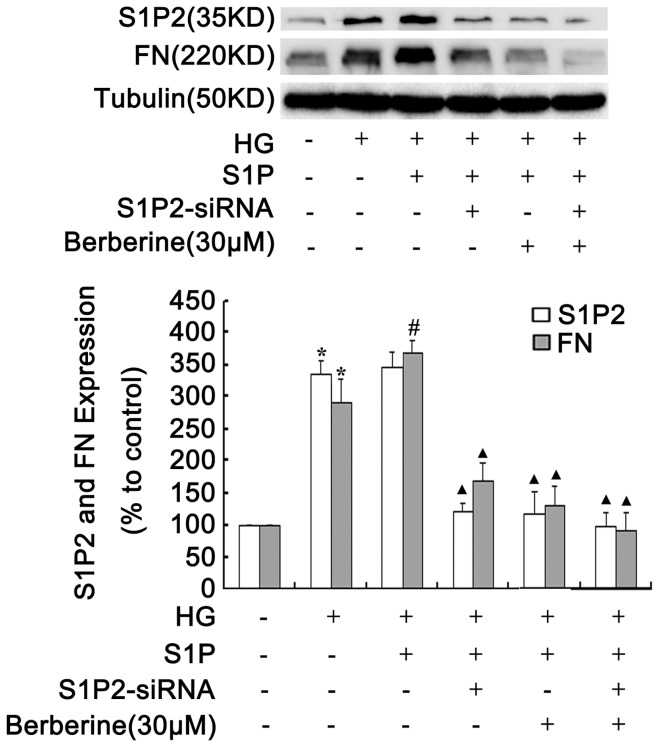
BBR suppressed S1P2 receptor-mediated FN expression in GMCs under HG condition. GMCs were treated with S1P, S1P2-siRNA and BBR conditionally under HG condition, and S1P2 receptor and FN protein expression were measured by Western blot assay. Data are represented as Means ± SDs, *P<0.05 vs. control, #*P*<0.05 vs. HG, ▴*P*<0.05 vs. S1P only.

Our previous studies have indicated that FN is reduced by BBR in GMCs under HG condition [5]. To explore whether the inhibitory effect of BBR on FN expression correlated with its influence on S1P2 receptor, specific siRNA of S1P2 receptor (S1P2-siRNA) was applied as a positive control. Fig. 4 showed that S1P2-siRNA significantly reversed the up-regulation of FN level induced by exogenous S1P in GMCs under HG condition (*P*<0.05), suggesting the involvement of S1P2 receptor in the increased FN expression under hyperglycemic condition. Similarly to S1P2-siRNA, BBR greatly down-regulated the S1P-S1P2 receptor-mediated FN level (*P*<0.05). After S1P2-siRNA pretreatment, BBR almost completely prevented S1P-S1P2 receptor-mediated FN expression (*P*<0.05, Fig. 4).

### BBR Inhibited NF-κB Activation Induced by HG in GMCs

The above-described experimental data suggested that BBR up-regulated the FN level by inhibiting S1P2 receptor expression. However, the underlying mechanism responsible for the influence of BBR on S1P2 receptor expression is still unclear. We have previously reported that BBR inhibited nuclear factor-kappa B (NF-**κ**B) activation and reduced ECM synthesis, thus ameliorating diabetic renal fibrosis [22], [23]. In view of this, the subsequent study was carried out to determine whether the down-regulation effects of BBR on S1P2 receptor expression was associated with its prevention of NF-**κ**B activation.

**Figure 5:**
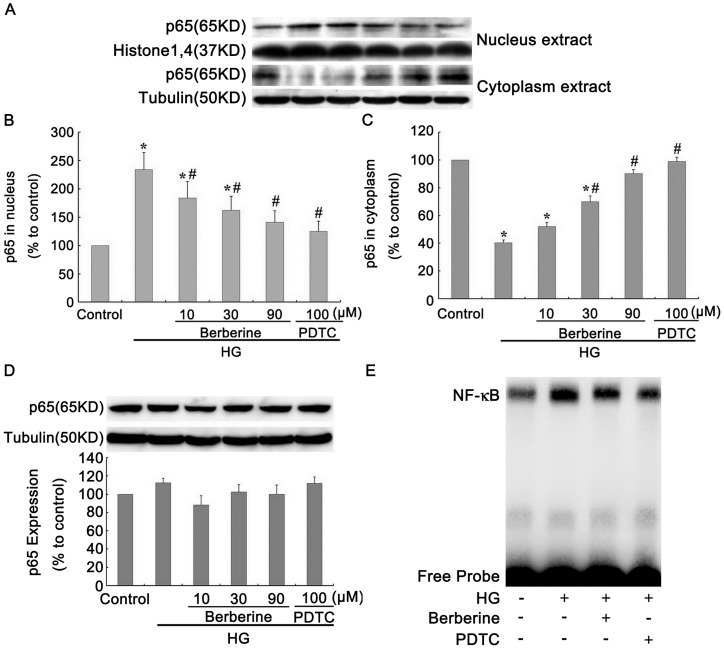
BBR inhibited NF-κB activation induced by HG in GMCs. GMCs were treated with BBR (10, 30, 90 µM) and PDTC (100 µM) for 24 h. Protein bands (A) were detected using the enhanced chemiluminescence detection system, p65 protein contents in nuclear (B), cytoplasm (C) and total p65 expression (D) were analyzed by Western Blot assay. (E) DNA binding activities of NF-**κ**B were determined by EMSA. Data are represented as Means ± SDs, *P<0.05 vs. control, #*P*<0.05 vs. HG.

Western blot assay was performed to detect the protein expression of p65 in the nucleus and cytoplasm, respectively. P65 protein is mainly expressed in the cytoplasm under normal condition. Compared with the control, HG treatment for 30 min resulted in significantly increased p65 contents in the nucleus and decreased p65 contents in the cytoplasm in GMCs (*P*<0.05, Figs. 5 B and 5 C). BBR and NF-**κ**B specific inhibitor pyrrolidine dithiocarbamate (PDTC, 100 µM; Sigma, USA) pretreatment for 2 h obviously decreased the p65 content in the nucleus and increased this content in the cytoplasm under hyperglycemia condition. The most significant effects were exerted by 90 µM BBR and 100 µM PDTC (*P*<0.05, Figs. 5 B and 5 C). This finding suggested that BBR had an inhibitory effect on NF-**κ**B nuclear translocation in a concentration-dependent manner under HG condition.

We also measured the total p65 protein expression under the same stimulus. Western blot assay data showed that the total p65 expression had no significant difference among all groups (*P*>0.05, Fig. 5 D), indicating that changes in the p65 content in the nucleus resulted from its translocation from the cytoplasm to the nucleus.

The effect of BBR on the DNA binding activity of NF-**κ**B was also evaluated by the electrophoretic mobility shift assay (EMSA). Compared with the control group, HG treatment for 2 h greatly increased the DNA binding activity of NF-**κ**B, whereas pretreatment with 30 µM BBR and PDTC for 2 h obviously decreased NF-**κ**B activity (Fig. 5 E).

### BBR and PDTC Suppressed S1P2 Receptor and FN Expression in GMCs under HG Condition

**Figure 6:**
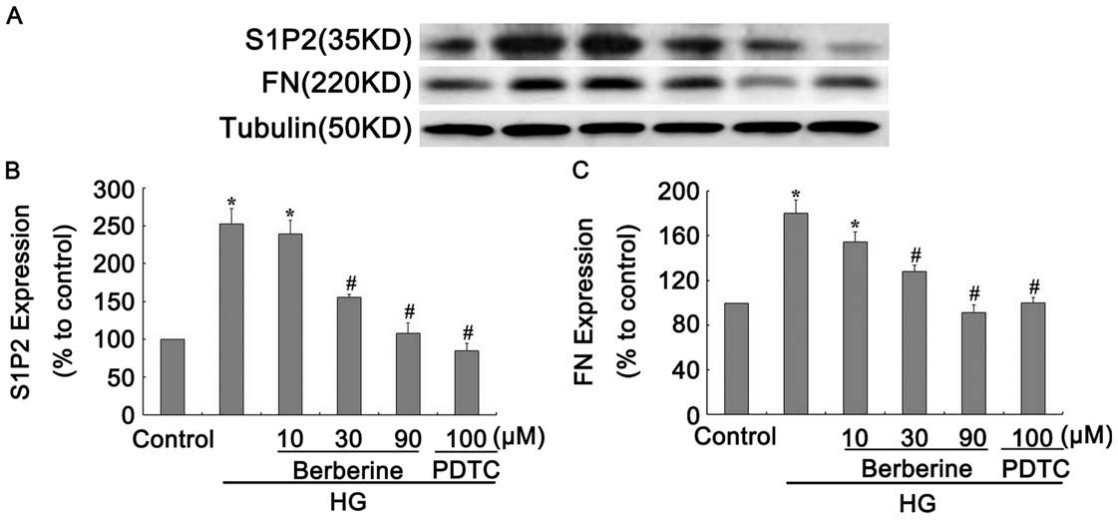
BBR and PDTC suppressed S1P2 receptor and FN expression in GMCs under HG condition. GMCs were treated with BBR (10, 30, 90 µM) and PDTC (100 µM) for 24h, protein bands (A) were detected using the enhanced chemiluminescence detection system, S1P2 receptor (B) and FN (C) expression were analyzed by Western Blot assay. Data are represented as Means ± SDs, *P<0.05 vs. control, #*P*<0.05 vs. HG.

The above data indicated that BBR inhibited NF-**κ**B activation, which was consistent with our previous studies. Therefore, we further investigated the relationship of the inhibitory effects of BBR on NF-**κ**B activation with the changes in S1P2 receptor and FN expression. Western blot results showed that PDTC obviously reduced the augmentation in S1P2 receptor and FN levels induced by HG (*P*<0.05, Figs. 6 B and 6 C). Similarly to PDTC, BBR also significantly reduced S1P2 receptor and FN protein expression (*P*<0.05, Figs. 6 B and 6 C). This finding suggested that the inhibitory effects of BBR on S1P2 receptor and FN levels might correlate with its influence on NF-**κ**B activation.

### S1P2-siRNA had no Effect on the Activation of NF-κB Pathway Induced by HG in GMCs

**Figure 7:**
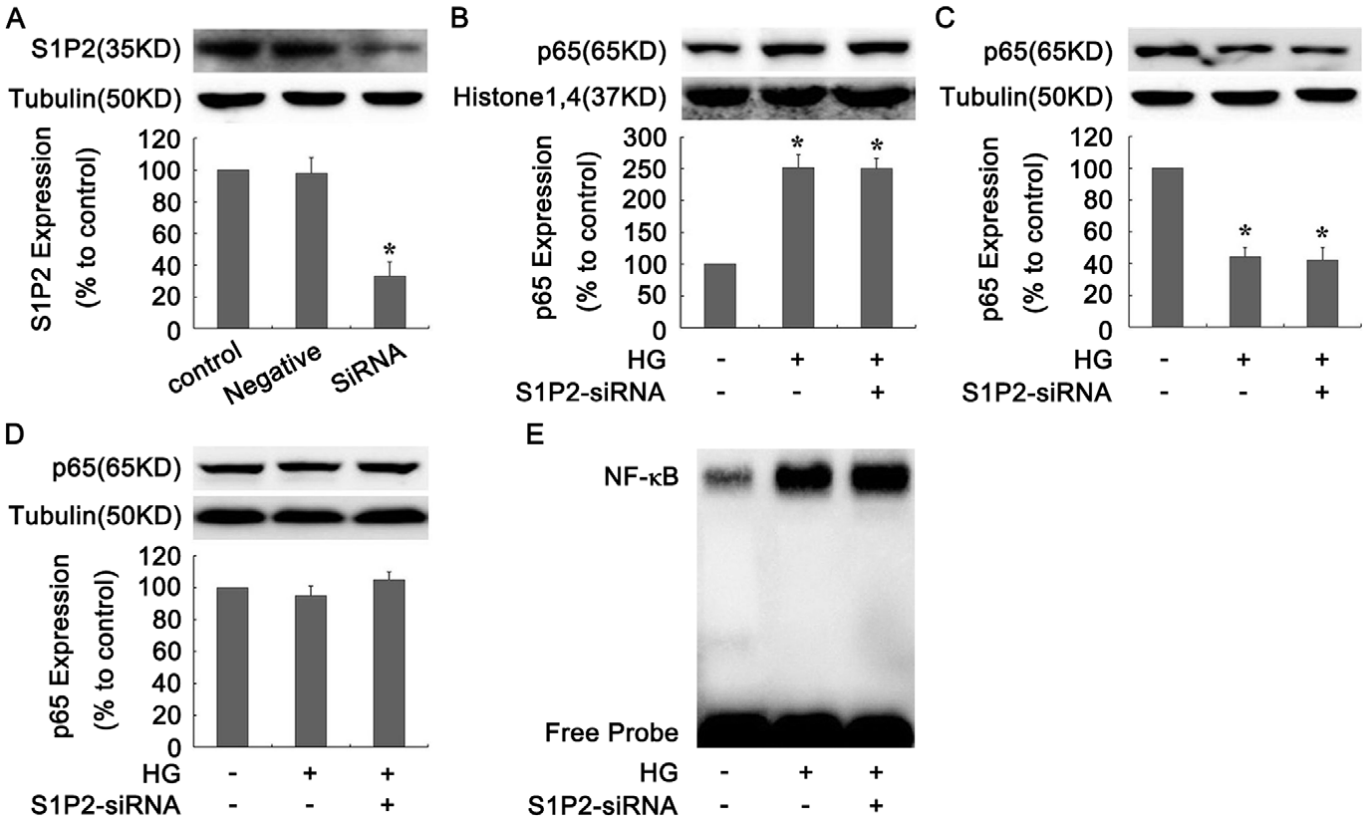
S1P2-siRNA had no effect on the activation of NF-κB pathway induced by HG in GMCs. GMCs were treated with HG under S1P2-siRNA pretreatment condition, The protein levels of S1P2 receptor (A), p65 protein contents in nuclear (B), cytoplasm (C) and total p65 expression (D) were analyzed by Western Blot assay in GMCs. (E) DNA binding activity of NF-**κ**B was determined by EMSA. Data are represented as Means ± SDs, *P<0.05 vs. control.

In order to confirm that NF-**κ**B regulation was truly upstream of the S1P2 receptor, we applied S1P2-siRNA as a control to carry out the same experiment as Fig. 5. As shown in Fig. 7, S1P2 receptor expression in protein level was significantly knockdown by S1P2-siRNA by ∼67% (*P*<0.05, Fig. 7 A). Silencing of S1P2 receptor had no obvious effect on the increase of p65 contents in the nucleus and decrease of it in the cytoplasm induced by HG in GMCs (*P*>0.05, Figs. 7 B and 7 C). Total p65 protein level under the same stimulus also had no significant difference among all groups (*P*>0.05, Fig. 7 D). And further EMSA result showed that the DNA binding activity of NF-**κ**B wasn’t affected by S1P2-siRNA (Fig. 7 E). All the above data showed that S1P2-siRNA didn’t affect the activation of NF-**κ**B pathway under HG condition, indicating that NF-**κ**B was truly the upstream of the S1P2 receptor and regulated the expression of S1P2 receptor.

## Discussion

STZ intraperitoneal injection was performed to create a diabetic renal dysfunction model in the current study, which is a well-established method by our research group. When the experiment was terminated on the 12th week, the renal hypertrophy index (KW/BW), BUN, Cr, and UP 24 h in diabetic rats were obviously enhanced compared with those in normal control. The BBR-treated group significantly decreased the FBG, renal hypertrophy index, BUN, Cr, and UP 24 h, consistent with our previous studies that BBR notably ameliorated diabetic renal dysfunction [22]–[24]. The anti-DN effects of BBR are synergetic ones via multiple factors, part of which is relate to its functions of lowering blood glucose, reducing oxidative stress, and the regulating of polyol pathway. Based on these studies, the current research further explored the relationship between the protective effects of BBR on DN and S1P2 receptor in the SphK1/S1P signaling pathway.

By binding to its natural ligands, the S1P receptors S1P1-S1P5 on the cell membrane can induce extensive biological effects including the regulation of Ca^2+^ concentration, as well as participation in cell proliferation, survival, differentiation, apoptosis, and migration. The S1P receptors also affect endothelial injury, inflammation, thrombosis, angiogenesis, and vascular injury by activating or inhibiting multiple signaling pathways [25], [26]. The S1P2 receptor is mainly involved in some physiological and pathological processes such as immunity, inflammation, tumor formation, and so on [27]. However, knowledge on its roles in diabetes and diabetic complications remains limited. The S1P2 receptor signaling pathway is reportedly involved in GMCs proliferation induced by S1P in DN research [28], [29]. Imasawa et al. [30] have shown that S1P signals are preferentially transmitted via the S1P2 rather than S1P1 receptor to regulate the pathogenesis of glomerular endothelial injuries in the glomeruli of rats with DN. This observation emphasizes the importance of an unbalanced S1P2/S1P1 ratio in the progression of DN. Our previous studies have revealed that the SphK1/S1P pathway is remarkably activated in the diabetic renal cortex fragments of rats and mice, as well as in GMCs exposed to HG. The S1P2 receptor, which is closely associated with FN overproduction, is also expressed predominantly among the S1P1-S1P5 receptors [16]–[18].

Consistent with our previous results, the present study also found that S1P2 receptor expression increased in both diabetic rat kidneys and GMCs exposed to HG. Interfering with the expression of S1P2 receptor reduced FN production, indicating that the involvement of S1P2 receptor in FN overproduction under hyperglycemic condition. BBR significantly suppressed S1P2 receptor expression in the above models of diabetes, and prevented its distribution on the membrane of GMCs under HG condition. Similarly to S1P2-siRNA, BBR effectively inhibited S1P-S1P2 receptor- mediated FN overexpression. After the pretreatment of S1P2-siRNA, BBR further reduced the expression of S1P2 receptor and S1P induced FN expression, which might be attributed to the superimposed inhibitory effects of S1P2-siRNA and BBR on S1P2 receptor. The above mentioned results suggested that BBR could reduce FN expression by inhibiting S1P2 receptor in GMCs under diabetic condition besides its interfering with SphK1 (expression and activity) and diminishing S1P production [17]. The inhibitory effects of BBR exerted on the S1P-S1P2 receptor signaling pathway might be one of the mechanisms accounting for its anti-DN actions.

S1P2 receptor majorly participates in pathological injuries, such as immunity, and inflammation, whereas inflammation is a critical characteristic of diabetic renal fibrosis [31]. Our previous findings have revealed that BBR inhibits specifically the activation of NF-**κ**B inflammatory signaling pathway to reverse the DN independently of its hypoglycemic effect [22]. Therefore, further investigations were conducted to determine whether the underlying molecular mechanisms that contributed to the inhibitory effects of BBR on S1P2 receptor were related to its prevention of NF-**κ**B activation.

The present study showed that BBR reduced p65 nuclear translocation and inhibited the DNA binding activity of NF-**κ**B in GMCs under HG condition, demonstrating again that BBR prevented the activation of NF-**κ**B. We also found that the NF-**κ**B specific inhibitor PDTC obviously decreased the HG-mediated increased expression of S1P2 receptor, indicating the involvement of NF-**κ**B activation in the HG induced increase in S1P2 receptor. Similarly to PDTC, BBR also significantly reduced the protein expression of S1P2 receptor and FN while inhibiting NF-**κ**B nuclear translocation, suggesting that the reduction effect of BBR on S1P2 receptor might closely associate with its inhibitory effects on NF-**κ**B activation.

**Figure 8:**
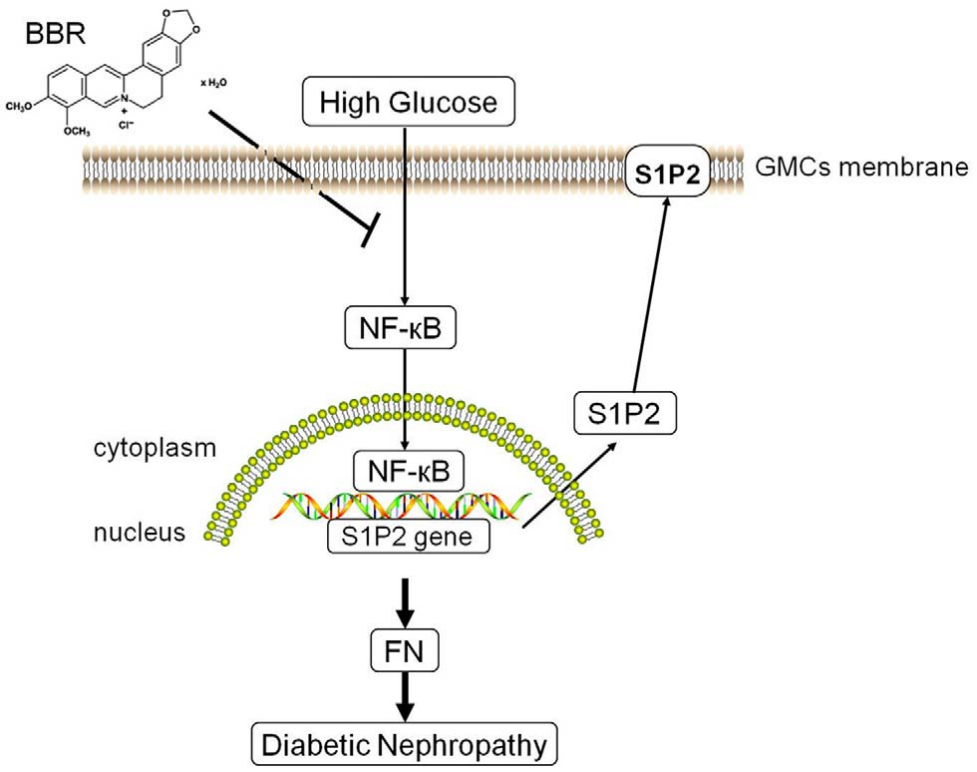
Schematic representation of the possible mechanism of BBR on the S1P2 receptor. Subjecting GMCs to HG results in the activation of NF-**κ**B signaling pathway, and thus up-regulates S1P2 receptor expression, which contributes to the increased FN levels. In contrast, pretreatment with BBR inhibits S1P2 receptor and FN levels possibly by down-regulating NF-**κ**B activation.

In conclusion, we found that BBR obviously decreased S1P2 receptor expression both in the kidneys of diabetic rat and GMCs exposed to HG. BBR also effectively inhibited the increase in FN levels induced by S1P2 receptor. The underlying molecular mechanism was closely correlated with its inhibitory effects on NF-**κ**B activation (Fig. 8). By initially identifying the molecular mechanisms responsible for the inhibitory effects of BBR on FN expression in the above diabetic models via the S1P2 receptor, we provided new experimental evidence on the anti-DN application of BBR via the SphK1/S1P/S1P2 receptor signaling pathway. However, the precise mechanism underlying the inhibition of NF-**κ**B activation induced by BBR and the subsequently influenced S1P2 receptor expression warrants further explorations.

## Materials and Methods

### Animal Experiment

All experimental procedures were carried out in accordance with the China Animal Welfare Legislation, and approved by the Ethics Committee on the Care and Use of Laboratory Animals of Sun Yat-sen University (permit number: 20100805003), Guangzhou, China. Healthy male Sprague Dawley rats weighing 210±20 g were obtained from the Medical Laboratory Animal Center, Guangdong Province, China. The rats were fasted overnight and then injected intraperitoneally once with 50 mg/kg STZ (Sigma Aldrich, USA) freshly dissolved in 10 mM sodium citrate buffer to induce diabetes. FBG levels were measured 72 h after STZ injection using a One-Touch glucometer (Johnson and Johnson, USA). Rats with FBG levels above 16.7 mM were considered diabetic. Diabetic rats were randomly divided into diabetic model (*n* = 10) and BBR treatment (*n* = 10) groups. Age-matched healthy rats were used as the normal control group (*n* = 10). The rats in the BBR treatment group were administered daily with 200 mg/kg BBR by gavage. The rats in both normal control and diabetic model groups were given the same volume of distilled water by gavage. Before the end of the experiment, urine was collected from the rats housed in metabolic cages for 24 h. At the end of the 12th week, all animals were sacrificed, blood samples were collected, and the serum was separated and stored at −20°C until use for biochemical analysis (mainly Cr and UP 24h). Kidney samples were quickly excised, weighed, frozen in liquid nitrogen, and stored at −80°C.

### Cell Culture

Sprague Dawley rat primary GMCs were isolated from glomeruli cortex fragments using standard protocols. Cells were grown in Dulbecco’s modified Eagle’s medium supplemented with penicillin-streptomycin and 10% fetal bovine serum (Gibco, USA) at 37°C in a 5% CO_2_ incubator. The subsequent experiments with GMCs were performed from Passages 3–12. GMCs were inactivated by serum deprivation for 24 h prior to treatment.

### RNA Isolation and Real-time Polymerase Chain Reaction (PCR)

Total RNA was isolated from cortex fragments of rat kidneys or GMCs using Trizol reagent according to the manufacturer’s instructions (Invitrogen, USA). RNA was precipitated with isopropanol and dissolved in diethylprocarbonate-treated water. Total RNA was then subjected to reverse transcription using a commercialized PrimeScript RT reagent kit (Takara, Japan), followed by quantitative real-time PCR using a Bio-Rad iCycler IQ system (Bio-Rad, USA). The specific primers used for the PCR amplifications were as follows: S1P2 receptor (forward: 5′-AAATCCAATACGGTA CAAACG-3′, reverse: 5′-GGTCAGACAGCACCCACA-3′), β-actin (forward: 5′-CCCATCTATGAG GGTTACGC-3′, reverse: 5′-TTTAATGTCACGCACGATTTC-3′). All reactions were conducted in triplicate.

### Small Interfering RNA (siRNA)

Validated Stealth™ negative control and double-stranded S1P2 receptor-specific siRNA oligonucleotides were synthesized by Ruibo (China). The following were the special sequences of S1P2 receptor-siRNA: sense: 5′-CAGGAACACUACAAUUACADTDT-3′, antisense: 5′-UGUAAUU GUAGUGUUCCUGDTDT-3′. GMCs were transfected for 6h with siRNA oligonucleotides using lipofectamine 2000 reagent (Invitrogen, USA) according to the instructions of the manufacturer. About 48 h after transfection, cells were harvested, and Western blot assay was performed to analyze the S1P2 receptor and FN protein levels.

### Western Blot Assay

Western blot assay was performed to detect the protein expression of S1P2 receptor, FN, and p65. Kidney cortex fragments or GMCs were lysed in radioimmunoprecipitation assay buffer (PH 8.0 50 mM Tris, 150 mM NaCl, 0.02% sodium azide, 0.1% SDS, 1% NP-40, 0.5% sodium deoxycholate, 1 mM EDTA, and so on) supplemented with phenylmethanesulfonyl fluoride (1mM; Sigma, USA) and protease inhibitor cocktail (100×; Calbiochem, USA). The membrane proteins of GMCs were extracted using an assay kit (Beyotime, China). Protein concentration was determined using a BCA™ Protein Assay Kit (Pierce, USA) following the protocol of the manufacturer. For the extraction of nuclear and cytoplasmic proteins of GMCs, a commercialized assay kit was used according to the protocol (**Nuclear protein extraction and electrophoretic mobility shift assay** subsection). Equal amounts of protein samples were separated by 10% SDS-PAGE and transferred onto a poly (vinylidene difluoride) membrane (Millipore, USA). Nonspecific binding was blocked with 5% non -fat dry milk at room temperature in TBST. After washing, the membranes were incubated overnight at 4°C with the following primary antibodies: rabbit polyclonal antibody against S1P2 receptor (1∶500; Cayman Chemical, USA; Bioworld, USA), mouse monoclonal antibody against FN and p65 (1∶1000; Santa Cruz Biotechnology, USA), mouse monoclonal antibody against Tubulin (1∶10 000; Sigma, USA), and mouse monoclonal antibody against Histone 1,4 (1∶1000; Sigma, USA). After further washing, the membranes were incubated for 1h with corresponding horseradish peroxidase- conjugated secondary antibodies (anti-rabbit IgG or anti-mouse IgG, 1∶10 000; Promega, USA). Immunoreactive bands were visualized using an enhanced chemiluminescence substrate (Pierce, USA) and captured on an X-ray film. The intensity of protein bands were quantitated using a Gel Doc XR System (Bio-Rad, USA) and analyzed by a UVP Gel Documentation System GDS8000 and Gel works Labwork 4.0 Analysis Software.

### LSCM

GMCs grown on glass coverslips were treated with the corresponding stimuli. Cells were washed, fixed in PBS with 4% paraformaldehyde for 15 min at room temperature, and permeabilized with Triton X-100 (1% in PBS) for 10 min at room temperature. After washing, cells were blocked with 10% goat serum for 45 min at room temperature and incubated with a rabbit monoclonal antibody against S1P2 receptor (1∶100 in 10% goat serum) overnight at 4°C. The cells were washed again and incubated in the dark with Alexa Fluor® 488-conjugated secondary antibody (1∶1000; Invitrogen, USA). Nuclei were co-labeled with Hoechst 33342 solution (5 µg/ml in PBS) for 10 min in the dark at room temperature. The coverslips were mounted on glass slides with anti-fade mounting media (Beyotime, China), and images were collected using a Zeiss LSM 510 laser confocal fluorescence microscope (CarlZeiss, Germany).

### Nuclear Protein Extraction and EMSA

The EMSA is a common technology used to investigate the interaction between DNA and protein or betwwen RNA and protein. The activation of NF-**κ**B was determined by the EMSA using nuclear extracts prepared by a Nuclear Extract Kit (Active Motif, USA) from different groups of cells. About 5.0×10^6^ cells were washed, collected with 2.5 ml of ice cold PBS/phosphatase inhibitors, and lysed in 250 µl of 1× hypotonic buffer. Subsequently, 25 µl of detergent was added and the cells were vortexed with maximal velocity for 10 s and centrifuged at 14 000* g* for 3 min at 4°C. After collecting the supernatant (cytoplasmic fraction), the pellets were resuspended in 25 µl of complete lysis buffer and centrifuged at 14 000* g* for 10min. The supernatants (nuclear proteins) were used for EMSA after determining the protein concentration using the Braford method (Qiangen, USA).

The sequence of the biotin-labeled oligonucleotide probes for NF-**κ**B (Beyotime, China) was as follows: 5′-AGTTGAGGGGACTTTCCCAGG-3; 3′-TCAACTCCCCTGAAAGGGTCCG-5′, containing the acknowledged NF-**κ**B binding site. The procedures were performed following the instructions of the manufacturer (Light Shift Chemiluminescent EMSA Kit; Pierce, USA). The nuclear proteins (3 µg) were incubated with 50 ng/µl poly (dI-dC), 0.05% Nonidet P-40, 5 mM MgCl_2,_ and 2.5% glycerol for 10 min. Subsequently, incubation at room temperature for 20 min with 0.2 pmol of biotin-labeled NF-**κ**B consensus oligonucleotide in a 12.5 µl volume was performed. The reaction mixture was then subjected to 7% non-denaturing SDS-PAGE, transferred onto a nylon hybridization transfer membrane (Amersham, USA), and DNA cross-linked for 10 min. After being blocked in a blocking buffer for 1h at room temperature, the blots was incubated with horseradish peroxidase-conjugated strept-avidin antibodies (1∶300) for 15 min. Peroxidase activity was detected using an enhanced chemiluminescence substrate system. The images were captured and quantified using Image Quant LAS 4000mini (GE Healthcare, USA).

### Statistical Analysis

Values were expressed as means ± SDs. All data were assessed by the SPSS 11.5 software. Unpaired Student’s t test was used for comparison between two groups. For multiple comparisons, data were analyzed by one-way ANOVA with post hoc multiple comparisons. Independent experiments were performed at least thrice with similar results. *P*<0.05 was considered statistically significant.

## References

[1] Schena FP, Gesualdo L (2005) Pathogenetic mechanisms of diabetic nephropathy. J Am Soc Nephrol 16: S30–S33. 15938030 10.1681/asn.2004110970

[2] Kanwar YS, Sun L, Xie P, Liu FY, Chen S (2011) A glimpse of various pathogenetic mechanisms of diabetic nephropathy. Annu Rev Pathol Mech Dis 6: 395–423. 10.1146/annurev.pathol.4.110807.092150 PMC3700379 21261520

[3] Jia HJ, Qi XD, Fang SH, Jin YH, Han XY, et al (2009) Carnosine inhibits high glucose-induced mesangial cell proliferation through mediating cell cycle progression. Regul Pept 154: 69–76. 19154760 10.1016/j.regpep.2008.12.004

[4] Wahab N, Cox D, Witherden A, Mason RM (2007) Connective tissue growth factor (CTGF) promotes activated mesangial cell survival via up-regulation of mitogen-activated protein kinase phosphatese -1 (MKP-1). Biochem J 406: 131–138. 17489738 10.1042/BJ20061817 PMC1948989

[5] Liu WH, Tang FT, Deng YH, Li XJ, Lan T, et al (2009) Berberine reduces fibronectin and collagen accumulation in rat glomerular mesangial cells cultured under high glucose condition. Mol Cell Biochem 325: 99–105. 19142714 10.1007/s11010-008-0024-y

[6] Giunti S, Barit D, Cooper ME (2006) Diabetic nephropathy: from mechanisms to rational therapies. Minerva Med 97: 241–262. 16855519

[7] Fyrst H, Saba JD (2010) An update on sphingosine-1-phosphate and other sphingolipid mediators. Nat Chem Biol 6: 489–497. 20559316 10.1038/nchembio.392 PMC3001344

[8] Alvarez SE, Milstien S, Spiegel S (2007) Autocrine and paracrine roles of sphingosine-1-phosphate. Trends endocrinol and Metab 18: 300–307. 17904858 10.1016/j.tem.2007.07.005

[9] Kihara A, Mitsutake S, Mizutani Y, Igarashi Y (2007) Metabolism and biological functions of two phosphorylated sphingolipids, sphingosine 1-phosphate and ceramide 1-phosphate. Prog Lipid Res 46: 126–144. 17449104 10.1016/j.plipres.2007.03.001

[10] Spiegel S, Milstien S (2003) Sphingosine-1-phosphate: an enigmatic signaling lipid. Nat Rev Mol Cell Biol 4: 397–407. 12728273 10.1038/nrm1103

[11] Ogretmen B, Hannun YA (2004) Biologically active sphingolipids in cancer pathogenesis and treatment. Nat Rev Cancer 4: 604–616. 15286740 10.1038/nrc1411

[12] Yatomi Y (2006) Sphingosine1-phosphate in vascular biology: possible therapeutic strategies to control vascular diseases. Curr Pharm Des 11: 575–587. 10.2174/138161206775474404 16472149

[13] Wang F, Okamoto Y, Inoki I, Yoshioka K, Du W, et al (2010) Sphingosine-1-phosphate receptor-2 deficiency leads to inhibition of macrophage proinflammatory activities and atherosclerosis in apoE-deficient mice. J Clin Invest 120: 3979–3995. 20978351 10.1172/JCI42315 PMC2964972

[14] Whetzel AM, Bolick DT, Srinivasan S, Macdonald TL, Morris MA, et al (2006) Sphingosine-1 phosphate prevents monocyte/endothelial interactions in type 1 diabetic NOD mice through activation of the S1P1 receptor. Circ Res 99: 731–739. 16960101 10.1161/01.RES.0000244088.33375.52

[15] Randriamboavonjy V, Badenhoop K, Schmidt H, Geisslinger G, Fisslthaler B, et al (2009) The S1P2 receptor expressed in human platelets is linked to the RhoA-Rho kinase pathway and is downregulated in type 2 diabetes. Basic Res Cardiol 104: 333–340. 19139947 10.1007/s00395-008-0769-1

[16] Lan T, Shen XY, Liu PQ, Liu WH, Xu SW, et al (2010) Berberine ameliorates renal injury in diabetic C57BL/6 mice: Involvement of suppression of SphK-S1P signaling pathway. Arch Biochem Biophys 502: 112–120. 20646989 10.1016/j.abb.2010.07.012

[17] Lan T, Liu WH, Xie X, Xu SW, Huang KP, et al (2011) Sphingosine kinase-1 pathway mediates high glucose-induced fibronectin expression in glomerular mesangial cells. Mol Endocrinol 25: 2094–2105. 21998146 10.1210/me.2011-0095 PMC3231833

[18] Liu WH, Lan T, Xie X, Huang KP, Peng J, et al (2012) S1P2 receptor mediates sphingosine -1- phosphate-induced fibronectin expression via MAPK signaling pathway in mesangial cells under high glucose condition. Exp Cell Res 318: 936–943. 22406263 10.1016/j.yexcr.2012.02.020

[19] Xia X, Yan JH, Shen YF, Tang KX, Yin J, et al (2011) Berberine Improves Glucose Metabolism in Diabetic Rats by Inhibition of Hepatic Gluconeogenesis. PLoS One 6: 1–10. 10.1371/journal.pone.0016556 PMC3033390 21304897

[20] Amasheh M, Fromm A, Krug SM, Amasheh S, Andres S, et al (2010) TNF alpha-induced and berberine-antagonized tight junction barrier impairment via tyrosine kinase. Akt and NF kappaB signaling J Cell Sci 123: 4145–4155. 21062898 10.1242/jcs.070896

[21] Kong W, Wei J, Abidi P, Lin M, Inaba S, et al (2004) Berberine is a novel cholesterol-lowering drug working through a unique mechanism distinct from statins. Nat Med 10: 1344–1351. 15531889 10.1038/nm1135

[22] Liu WH, Zhang XY, Liu PQ, Shen XY, Lan T, et al (2010) Effects of berberine on matrix accumulation and NF-kappa B signal pathway in alloxan-induced diabetic mice with renal injury. Eur J Pharmacol 638: 150–155. 20447389 10.1016/j.ejphar.2010.04.033

[23] Jiang Q, Liu PQ, Wu XQ, Liu WH, Shen XY, et al (2011) Berberine attenuates lipopolysaccharide -induced extracelluar matrix accumulation and inflammation in rat mesangial cells: involvement of NF-κB signaling pathway. Mol Cell Endocrinol 331: 34–40. 20674665 10.1016/j.mce.2010.07.023

[24] Liu WH, Liu PQ, Tao S, Deng YH, Li XJ, et al (2008) Berberine inhibits aldose reductase and oxidative stress in rat mesangial cells cultured under high glucose. Arch Biochem Biophys 475: 128–134. 18471986 10.1016/j.abb.2008.04.022

[25] Olivera A, Rosenfeldt HM, Bektas M, Wang F, Ishii I, et al (2003) Sphingosine kinase type 1 induces G12/13-mediated stress fiber formation, yet promotes growth and survival independent of G protein-coupled receptors. J Biol Chem 278: 46452–46460. 12963721 10.1074/jbc.M308749200

[26] English D, Garcia JG, Brindley DN (2001) Platelet-released phospholipids link haemostasis and angiogenesis. Cardiovasc Res 49: 588–599. 11166272 10.1016/s0008-6363(00)00230-3

[27] Aarthi JJ, Darendeliler MA, Pushparaj PN (2011) Dissecting the role of the S1P/S1PR axis in health and disease. J Dent Res 90: 841–854. 21248363 10.1177/0022034510389178

[28] Hanafusa N, Yatomi Y, Yamada K, Hori Y, Nangaku M, et al (2002) Sphingosine 1-phosphate stimulates rat mesangial cell proliferation from outside the cells. Nephrol Dial Transplant 17: 580–586. 11917049 10.1093/ndt/17.4.580

[29] Katsuma S, Ruike Y, Yano T, Kimura M, Hirasawa A, et al (2005) Transcriptional regulation of connective tissue growth factor by sphingosine 1-phosphate in rat cultured mesangial cells. FEBS Lett 579: 2576–2582. 15862293 10.1016/j.febslet.2005.03.073

[30] Imasawa T, Kitamura H, Ohkawa R, Satoh Y, Miyashita A, et al (2010) Unbalanced expression of sphingosine 1-phosphate receptors in diabetic nephropathy. Exp Toxicol Pathol 62: 53–60. 19261455 10.1016/j.etp.2009.02.068

[31] Navarro González JF, Mora Fernández C, Muros de Fuentes M, García Pérez J (2011) Inflammatory molecules and pathways in the pathogenesis of diabetic nephropathy. Nat Rev Nephrol 7: 327–340. 21537349 10.1038/nrneph.2011.51

