# Correction: Berberine Reduces Fibronectin Expression by Suppressing the S1P-S1P2 Receptor Pathway in Experimental Diabetic Nephropathy Models

**DOI:** 10.1371/annotation/e4e8ca0c-f6e8-4b32-aae1-b5f8e0c7ebc3

**Published:** 2012-10-25

**Authors:** Kaipeng Huang, Weihua Liu, Tian Lan, Xi Xie, Jing Peng, Juan Huang, Shaogui Wang, Xiaoyan Shen, Peiqing Liu, Heqing Huang

There were errors in the Funding section. The correct funding information is as follows: This work was supported by research grants from the National Natural Science Foundation of China (No. 81170676), Guangdong Major Science and Technology Project (No. 2011A080502004), Guangzhou Science and Technology Project (No. 10A32060084) and the Key Project of Guangdong Natural Science Foundation (NO. S2012020010991). The funders had no role in study design, data collection and analysis, decision to publish, or preparation of manuscript.

